# Depressive rumination and trait anxiety mediate the effects of childhood victimization on adulthood depressive symptoms in adult volunteers

**DOI:** 10.1371/journal.pone.0286126

**Published:** 2023-05-23

**Authors:** Jiro Masuya, Chihiro Morishita, Motoki Higashiyama, Ayaka Deguchi, Yoshitaka Ishii, Miki Ono, Mina Honyashiki, Yoshio Iwata, Takeshi Inoue

**Affiliations:** Department of Psychiatry, Tokyo Medical University, Shinjuku-ku, Tokyo, Japan; University of Concepcion Faculty of Medicine: Universidad de Concepcion Facultad de Medicina, CHILE

## Abstract

**Background:**

Prior studies have reported that childhood victimization experiences substantially augment the risk of depression and suicide in adulthood. Several of our previous studies suggested that childhood experiences of victimization interact with the quality of parenting experienced in childhood, childhood experiences of abuse, neuroticism, and other factors to influence depressive symptoms in adulthood. In this study, it was hypothesized that “childhood victimization” worsens “trait anxiety” and “depressive rumination”, and that “trait anxiety” and “depressive rumination” are mediators that worsen “depressive symptoms in adulthood”.

**Subjects and methods:**

The following self-administered questionnaires were completed by 576 adult volunteers: Patient Health Questionnaire-9, State–Trait Anxiety Inventory form Y, Ruminative Responses Scale, and Childhood Victimization Rating Scale. Statistical analyses were performed by Pearson correlation coefficient analysis, t-test, multiple regression analysis, path analysis, and covariance structure analysis.

**Results:**

Path analysis demonstrated that the direct effect was statistically significant for the paths from childhood victimization to trait anxiety, depressive rumination, and depressive symptom severity. Moreover, the indirect effect of childhood victimization on depressive rumination mediated by trait anxiety was statistically significant. The indirect effects of childhood victimization on depressive symptom severity mediated by trait anxiety and depressive rumination were statistically significant. Furthermore, the indirect effect of childhood victimization on depressive symptom severity mediated by both trait anxiety and depressive rumination was statistically significant.

**Conclusions:**

We found that childhood victimization directly and adversely influenced each of the above factors, and indirectly worsened adulthood depressive symptoms with trait anxiety and depressive ruminations as mediating factors. The present study is the first to clarify these mediation effects. Therefore, the results of this study suggest the importance of preventing childhood victimization and the importance of identifying and addressing childhood victimization in patients with clinical depression.

## Introduction

Previous studies have reported that the experience of childhood peer victimization, which is a different concept from childhood abuse, and is caused by negative behaviors of school and community members, significantly increases the risk of depression and suicide-associated problems in adulthood [1–10]. To our knowledge, mediating factors in the association between childhood victimization and mental illness have not been reported to date, other than in our 4 previous studies on adult community volunteers, as outlined below. First, we found that the experience of childhood victimization influenced depressive symptoms in adulthood, and this effect was mediated by neuroticism [11]. Second, we found that childhood experiences of abuse influenced depressive symptoms in adulthood, and this effect was mediated by childhood victimization and neuroticism [12]. Third, we found that the quality of parenting experienced in childhood influenced depressive symptoms in adulthood, and this effect was mediated by childhood victimization and negative life events in adulthood [13]. Finally, we found that childhood experiences of victimization are a risk factor for presenteeism in adulthood, and this effect is mediated by neuroticism and adverse effects on work stressors [14]. Our series of studies suggested that childhood victimization interacts with childhood parenting, childhood abuse, neuroticism, and stressors to impact depression. However, the role of other individual factors associated with depression, such as trait anxiety and rumination, on childhood victimization remains unclear [15, 16].

On the other hand, depressive rumination has been focused on as a factor associated with depressive symptoms, anxiety symptoms and major depression [17–20]. Rumination in a broad sense is defined as repetitive thoughts regarding past events and emotions, whereas depressive rumination is defined as repetitive thoughts and behaviors that focus attention on one’s depressive symptoms and the causes and effects of the symptoms [17]. Subjects with high depressive rumination reportedly have a higher incidence of major depression [17–20]. Depressive rumination not only increases and prolongs depression, anxiety, and stress, but has also been indicated to be a significant risk factor for the development of depression [17–20].

We previously reported that childhood experiences of abuse worsened adulthood depressive symptoms through the mediation effects of depressive rumination and trait anxiety, and that trait anxiety worsened depressive symptoms in adulthood not only by a direct effect but also by an indirect effect through depressive rumination as a mediator [16]. Another study also reported that depressive rumination is a mediator between childhood abuse and depression/anxiety in nonclinical adult volunteers [21]. On the other hand, the experience of childhood victimization, which is different from childhood abuse and is similar to bullying, reportedly worsened trait anxiety during college, around age 20 [22]. However, the effects of childhood victimization, such as harassment, on depressive rumination, and whether trait anxiety and depressive rumination mediate the effects of childhood victimization on adulthood depressive symptoms have not been investigated to date.

Based on the above theoretical model, we hypothesized and tested the hypothesis that “childhood victimization” has an adverse influence on “trait anxiety” and “depressive rumination”, which in turn have an adverse influence on “depressive symptoms in adulthood” as mediating factors for an indirect effect. We chose covariance structure analysis as our method to analyze the interrelationships and mediating effects of the following 4 factors: victimization in childhood, and trait anxiety, depressive rumination, and depressive symptoms in adulthood. This was a cross-sectional study in which questionnaires were administered to adult volunteers regarding the above 4 factors.

### Subjects and methods

#### Subjects

This study was part of a large-scale questionnaire survey on adult volunteers regarding their demographics, personality, stress, sleep, affective symptoms, happiness, resilience, and quality of life [12–14, 16]. Between April 2017 and April 2018, the questionnaires and instructions for this study were distributed by convenience sampling to 1,237 adults in the community through our acquaintances at Tokyo Medical University. The study was conducted using paper-based questionnaires. The inclusion criteria were being 20 years or older, and agreeing to participate. The exclusion criteria were having a severe physical disease or an organic mental disease. The researchers disclosed that participation was completely voluntary, and that nonparticipation would not result in any disadvantages. Data were anonymized to prevent any external disclosure of personal information. A total of 597 adult volunteers participated after providing informed consent (48.3%), and finally 576 volunteers were included in the analysis (men: 249; women: 327; average age: 41.6 ± 12.0 years) after excluding those who returned questionnaires with missing data. All subjects provided written informed consent to participate in this study. The items used for analysis were demographic information, childhood victimization, trait anxiety, depressive rumination, and depressive symptoms. In this study we defined childhood victimization as general childhood victimization caused by negative behaviors from school and community members, which is more broad than so-called childhood bullying [11]. In addition, this study did not take into account whether childhood victimization was intentional or not, nor whether there was a power imbalance. The reason for this is that the definition of childhood bullying that is commonly used internationally is the one by Olweus, namely “repeated physical and psychological acts by a student (or group of students) who is considered strong against a student who is considered weak” [23]. Three types of childhood victimization are analyzed in this study, namely, physical victimization, verbal victimization, and relational victimization by school and/or community members. This study was conducted in accordance with the 1964 Declaration of Helsinki (amended in Fortaleza in 2013), and was approved by the Institutional Review Board of Tokyo Medical University (study approval no.: SH3502).

### Questionnaires

#### Patient Health Questionnaire-9 (PHQ-9)

The PHQ-9 is a self-report assessment scale to measure the severity of the symptoms of depression [24, 25]. In this study, the PHQ-9 summary score, which is the sum of the 9 items rated on a 4-point Likert scale, with higher scores indicating more severe depressive symptoms, was used. The Cronbach’s alpha for the total score of this scale was 0.854, indicating good internal consistency.

#### Ruminative Responses Scale (RRS)

The RRS is a self-administered questionnaire that includes 22 items evaluated on a 4-point Likert scale to measure the frequency of depressive rumination [26]. The Japanese version used in this study has been confirmed to be reliable and valid [27]. The total score for 22 items were analyzed. The Cronbach’s alpha for the total score of this scale was 0.944, indicating excellent internal consistency.

#### State–Trait Anxiety Inventory form Y (STAI-Y)

STAI-Y is a self-administered scale that measures state and trait anxiety [28]. Specifically, this study focused on trait anxiety, which reflects a person’s own anxiety-prone nature that is minimally influenced by anxiety-provoking situations. This study uses 20 items on trait anxiety, which are rated using a 4-point scale. In this study, the Japanese version developed and validated by Hidano et al. was used [29]. The Cronbach’s alpha for the total score of the trait anxiety subscale was 0.925, indicating excellent internal consistency.

#### Childhood Victimization Rating Scale

This study also used a revised version of the Childhood Victimization Rating Scale published by the National Institute for Educational Policy Research in Japan [11, 12]. The score of this scale was reported to significantly correlate with neuroticism and depressive symptom severity [11, 12]. This scale is a self-administered questionnaire on the degree and frequency of victimization in local communities or schools during childhood. The items on this scale consist of a 5-point Likert scale. In this study, the total score of 5 items was used in the analysis. Higher total scores indicate more severe childhood victimization. The Cronbach’s alpha for the total score of this scale was 0.862, indicating good internal consistency.

### Data analysis

Statistical analyses were performed by the *t*-test or Pearson’s correlation coefficient using SPSS statistics for Windows version 28 software (IBM, Armonk, NY, USA). Multiple regression analysis using the forced entry method was conducted with the PHQ-9 score as the dependent variable and the demographic information and questionnaire scores as independent variables. Next, the mediating effects and interrelationships among the variables were analyzed by path analysis. The comparative fit index (CFI) and root mean square error of approximation (RMSEA) were used as goodness-of-fit indices in the path analysis in this study. A CFI of 0.97 or higher and an RMSEA of 0.05 or lower were considered as good fits [30]. Covariance structure analysis with the robust maximum likelihood estimation (Mplus version 8.4, Muthén & Muthén, Los Angeles, CA, USA) was used for the overall judgment. All coefficients of the structural analysis of covariance were standardized for the analysis of direct and indirect effects. A *p*-value of less than 0.05 was considered statistically significant. For the path analysis, imputation methods of Mplus 8.4 software were used for the missing data in this study.

## Results

### Demographic information and depressive symptom (PHQ-9), trait anxiety (STAI-Y), depressive rumination (RRS), and childhood victimization (Childhood Victimization Rating Scale) scores of the participants (Tables 1 and 2)

Table 1 shows the demographic data and scores for PHQ-9, STAI-Y, RRS, and Childhood Victimization Rating Scale of the 576 participants. Table 2 shows the results of analysis of the association between each parameter and depressive symptoms, as indicated by the PHQ-9 using the *t*-test or Pearson’s correlation coefficient (*r*). Women, and those who were unmarried, childless, living alone, and with a history of or currently having a mental illness had significantly higher PHQ-9 scores. Age, years of education, employment status, and physical illness were not associated with PHQ-9 scores. STAI-Y score, RSS score, and Childhood Victimization Rating Scale score were significantly positively correlated with the severity of depressive symptoms.

**Table 1:** Demographic characteristics, and PHQ-9, RRS, STAI-Y, and victimization scores of the subjects.

Characteristic or measure	Number or mean ± SD
Age (years)	41.6 ± 12.0
Sex (men: women)	249: 327
Years of education	14.6 ± 1.8
Employment status (employed: non-employed)	562: 10
Current marital status (married: single)	377: 194
Presence of offspring (yes: no)	358: 214
Living alone (yes: no)	114: 453
Current physical disease (yes: no)	111: 465
Past history of mental illness (yes: no)	68: 508
Current mental illness (yes: no)	23: 544
PHQ-9 summary score	4.1 ± 4.3
RRS total score	35.2 ± 11.4
STAI-Y score (trait anxiety)	43.0 ± 10.4
Victimization rating scale score	2.4 ± 3.3

Data are presented as means ± standard deviations (SD) or numbers

PHQ-9, Patient Health Questionnaire-9; RRS, Ruminative Responses Scale; STAI-Y, State-Trait Anxiety Inventory Form Y

**Table 2:** Correlation of characteristics with PHQ-9 score (*r*) or effects on PHQ-9 summary score.

Characteristic or measure	Correlation with PHQ-9 summary score (*r*) or effect on PHQ-9 summary score (mean ± SD, *t*-test)
Age (years)	*r* = –0.032, *p* = 0.448
Sex (men: women)	Men (3.5 ± 4.1) vs women (4.5 ± 4.3), *p* = 0.005 (*t*-test)
Years of education	*r* = –0.073, *p* = 0.078
Employment status (employed: non-employed)	Employed (4.0 ± 4.2) vs non-employed (3.1 ± 4.9), *p* = 0.48 (*t*-test)
Current marital status (married: single)	Married (3.5 ± 3.9) vs single (5.2 ± 4.7), *p* < 0.000 (*t*-test)
Presence of offspring (yes: no)	Yes (3.8 ± 4.1) vs no (4.6 ± 4.5), *p* = 0.036 (*t*-test)
Living alone (yes: no)	Yes (5.1 ± 4.7) vs no (3.8 ± 4.1), *p* = 0.011 (*t*-test)
Current physical disease (yes: no)	Yes (4.2 ± 4.6) vs no (4.0 ± 4.2), *p* = 0.651 (*t*-test)
Past history of mental illness (yes: no)	Yes (6.7 ± 5.4) vs no (3.7 ± 4.0), *p* < 0.001 (*t*-test)
Current mental illness (yes: no)	Yes (7.8 ± 4.9) vs no (3.9 ± 4.2), *p* < 0.001 (*t*-test)
RRS total score	*r* = 0.499, *p* < 0.001
STAI-Y score (trait anxiety)	*r* = 0.639, *p* < 0.001
Victimization rating scale score	*r* = 0.277, *p* < 0.001

Data are presented as means ± standard deviations (SD) or numbers. *r* = Pearson’s correlation coefficient

PHQ-9, Patient Health Questionnaire-9; RRS, Ruminative Responses Scale; STAI-Y, State-Trait Anxiety Inventory Form Y

### Multiple regression analysis for PHQ-9 summary scores (Table 3)

Table 3 presents the results of multiple regression analysis for PHQ-9 summary scores. We analyzed which factors (i.e., childhood victimization, trait anxiety, depressive rumination, and several demographic factors) had a significant effect on depressive symptoms. In multiple regression analysis, total scores of each of the victimization scale, RRS, and STAI-Y trait anxiety were used as independent variables. Ten other factors, including age, sex, living alone, presence of children, marital status, employment status, years of education, current physical illness, current mental illness, and past history of mental illness, were also analyzed for their association with depressive symptoms, and the results are shown in Table 2. The forced entry method was used for the analysis. The results indicate that the scores for STAI-Y trait anxiety, RRS, and victimization scale were significantly associated with depressive symptoms. No statistically significant associations were found between all other factors and depressive symptoms. The adjusted *R*^2^ was 0.459, and the model explained 45.9% of the variance in depressive symptoms. No multicollinearity was found.

**Table 3:** Results of multiple regression analysis of PHQ-9 summary scores.

Independent variable	β	*p*-value	VIF
STAI-Y score (trait anxiety)	0.506	< 0.001	1.552
RRS total score	0.167	< 0.001	1.645
Victimization score	0.091	0.007	1.160
Living alone	0.073	0.067	1.607
Sex	0.065	0.057	1.167
Past history of mental illness	0.062	0.084	1.307
Current mental illness	0.050	0.154	1.257
Presence of offspring	0.024	0.593	1.998
Age	0.022	0.613	1.947
Years of education	−0.021	0.568	1.397
Current marital status	0.010	0.814	1.971
Employment status	−0.006	0.845	1.031
Current physical disease	−0.004	0.913	1.278

β, standardized partial regression coefficient; VIF, variance inflation factor

Dependent variable: PHQ-9 summary score

Independent variables: age, sex (men = 0, women = 1), marital status (single = 0, married = 1), presence of offspring (no = 0, yes = 1), living alone (no = 0, yes = 1), education years, employment status (non-employed = 0, employed = 1), current physical disease (absence = 0, presence = 1), past history of mental illness (absence = 0, presence = 1), current mental illness (absence = 0, presence = 1), STAI-Y trait anxiety score, RRS total score, victimization rating scale score

Adjusted *R*^2^ = 0.459; *F* = 36.496; *p* < 0.001

### Path analysis (Figs 1 and 2)

**Fig 1:**
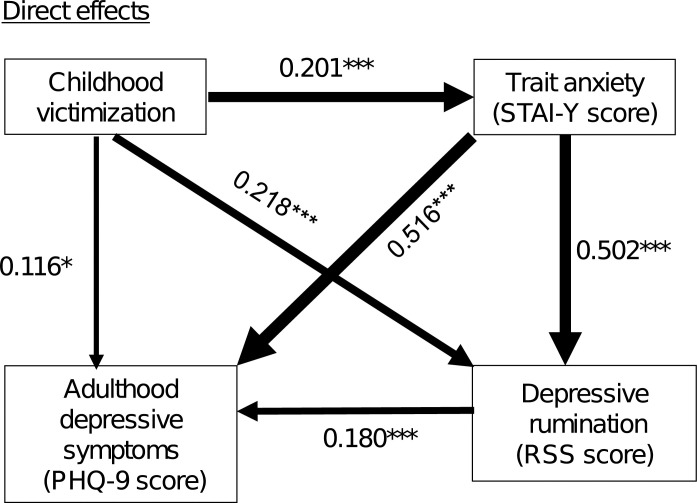
Results of the direct effects identified by path analysis between the 4 factors (childhood victimization, and trait anxiety, depressive rumination, and depressive symptoms in adulthood) in 576 adult volunteers from the community. The severity of depressive symptoms assessed by the PHQ-9 was the dependent variable. The solid arrows indicate statistically significant paths. The larger the statistically significant effect, the thicker the arrow. The numbers beside the arrows indicate the standardized path coefficients. *R*^2^ = 0.451, *p < 0.05, ***p < 0.001.

**Fig 2:**
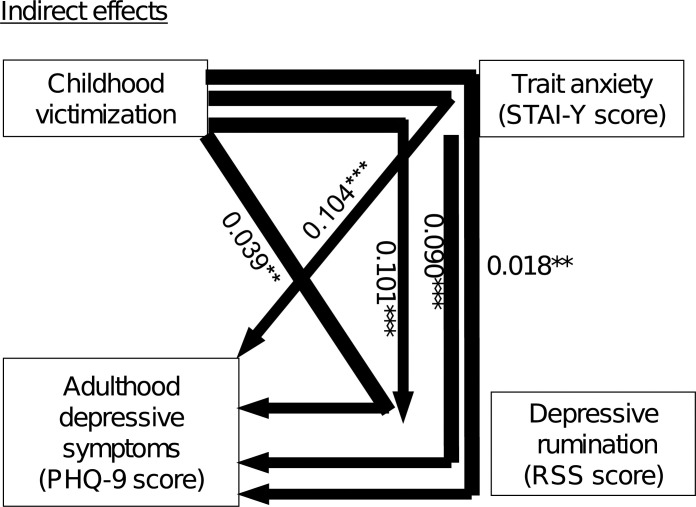
Results of the indirect effects identified by path analysis between the 4 factors (childhood victimization, trait anxiety, depressive rumination, and depressive symptoms in adulthood) in 576 adult volunteers from the community. The severity of depressive symptoms assessed by the PHQ-9 was the dependent variable. The solid arrows indicate statistically significant paths. The larger the statistically significant effect, the thicker the arrow. The numbers beside the arrows show the standardized path coefficients. *R*^2^ = 0.454, **p < 0.01, ***p < 0.001.

Figs 1 and 2 show the results of path analysis with depressive symptom severity as the dependent variable. The goodness-of-fit indices for the path model were CFI = 1.000 and RMSEA = 0.000, indicating that the model fit was adequate [30]. Fig 1 shows the direct effects, which were statistically significant for the paths from childhood victimization to trait anxiety, depressive rumination, and depressive symptom severity. Moreover, statistically significant direct effects were found for the paths from trait anxiety to depressive rumination and to depressive symptom severity, and from depressive rumination to depressive symptom severity. The *R*^2^ was 0.451; i.e., this model explains 45.1% of the variation of depressive symptom severity among the adult volunteers.

Fig 2 illustrates the indirect effects of the path analysis. The impact of childhood victimization on depressive rumination mediated by trait anxiety was statistically significant (standardized coefficient β = 0.101, *p* < 0.001). The impact of trait anxiety on the severity of depressive symptoms mediated by depressive rumination was statistically significant (β = 0.090, *p* < 0.001). Furthermore, the impacts of childhood victimization on depressive symptom severity mediated by trait anxiety and depressive rumination were statistically significant (β = 0.104, *p* < 0.001 and β = 0.039, *p* < 0.01, respectively). Moreover, the impact of childhood victimization on the severity of depressive symptoms mediated by both trait anxiety and depressive rumination was statistically significant (β = 0.018, *p* < 0.01).

## Discussion

In the present study, we analyzed the interrelationships and mediating effects of 4 factors, namely, childhood victimization, and trait anxiety, depressive rumination, and depressive symptoms in adulthood, and found that childhood victimization had a direct and adverse effect on each of the other 3 factors, and had an indirect and adverse effect on depressive symptoms in adulthood through trait anxiety and depressive rumination as mediating factors. To our knowledge, the present study is the first to clarify these mediation effects, and the results are partly consistent with previous findings, including our own [11–14, 16].

It has been reported that childhood victimization influences the development of depressive symptoms and major depression in adulthood, and that these effects are mediated by neuroticism [11, 12]. In addition, in another previous study we reported that the experience of being abused in childhood worsened depressive symptoms, which was mediated by trait anxiety and depressive rumination [16]. In addition, Storch et al. (2004) reported the adverse effect of victimization in childhood on trait anxiety during university [22]. The results of these previous studies and the present study are in good agreement. Our present study partially reconfirmed our previous findings [11, 16]. It is known that childhood experiences of victimization, like childhood experiences of abuse, have a significant effect on a person’s psychological development, but the mechanism by which this occurs is not well understood [1–10]. The present results suggest that trait anxiety and depressive rumination play a role in the mechanism of the effects of childhood victimization experiences on a person’s psychological development and mental health in adulthood.

When faced with an event that is difficult to accept, people have a tendency to avoid it (i.e., suppression) [31]. However, an experimental study demonstrated that suppressing thoughts on a particular subject or diverting focus onto a different subject paradoxically activates those thoughts [32]. Moreover, a paradoxical process of thought suppression has been suggested, i.e., as long as a people try to suppress thoughts about a particular topic, they will continue to think about it [33]. Several reports have discussed the association between this paradoxical effect of thought suppression and depressive rumination [34, 35]. Rumination is a product of thought suppression and the paradoxical phenomenon of increased negative thinking observed after attempts to suppress negative thoughts [34, 35]. Furthermore, a study on the influence of interactions between thought suppression and stressors on depressive rumination among university students found that under high stress, the group with stronger thought suppression was more prone to depressive rumination than the group with weaker thought suppression [35]. This finding indicates that thought suppression may be a major factor in depressive rumination. The experience of childhood victimization is difficult to accept, but suppressing thoughts on childhood victimization may paradoxically activate those thoughts and amplify negative depressive rumination that stems from childhood victimization, which may provoke long-term negative effects, such as depressive symptoms. The results of the present study are in agreement with this hypothesis.

Burden on the developing brain, such as the experience of adversities in childhood has been pointed out as a cause of depression and other mental disorders [36, 37]. Stressful experiences, such as being abused or bullied, lead to the oversecretion of cortisol, resulting in damage to the brain [38–40]. Specific parts of the brain are significantly more atrophied in those who experienced bullying victimization and/or abuse in childhood [41–43]. On the other hand, a positive association was observed between trait anxiety and cortisol secretion [44]. Furthermore, a synergistic effect of the functional connectivity between the basolateral nucleus of the amygdala and the pregenual anterior cingulate cortex in the brain on the stress hormones cortisol and norepinephrine has been suggested to be involved in the mechanism facilitating the recall of negatively biased memories that underlies depressive rumination [45]. Therefore, the experience of childhood victimization may induce the oversecretion of cortisol, which damages the brain and weakens its function, triggering depressive rumination and trait anxiety. Thus, these biological processes, together with childhood victimization, trait anxiety, and depressive rumination, may influence the development of adulthood depressive symptoms, as indicated in the present study.

It has been noted that intervening and investing in the health, education, and welfare sectors in early childhood brings positive benefits to children, families, and the broader community, and ultimately these benefits lead to increased national productivity and gross domestic product [46]. Our recent study reported that childhood victimization experiences exacerbate presenteeism in adulthood, with adverse effects on neuroticism and work stressors as mediating factors [14]. The findings of the present study suggest that the mediating effects of trait anxiety and depressive rumination on adulthood depressive symptoms may be targets for the development of interventions in childhood that will produce long-term positive economic outcomes.

There are several limitations to this study. First, because it was a questionnaire survey that was based in part on the participants’ past memories, memory bias may have influenced the results. Second, many of the participants were healthy adult volunteers, and hence the results of this study may not be applicable to patients with clinical depression. Third, the cross-sectional nature of this study limits causal conclusions. Future long-term prospective studies are needed to confirm the associations found in the present study.

## Conclusion

The present results suggest that people who have experienced victimization in childhood are more likely to have trait anxiety, which is apt to produce anxiety symptoms, leading to depressive rumination. Depressive rumination, which is having repeating thoughts of past adverse events, acts as a mediating factor in the worsening of depressive symptoms during adulthood. These results are clinically meaningful because they will assist towards providing appropriate treatment and support to patients, such as introducing trauma care and cognitive behavioral therapy, by assessing the presence/absence of the experience of victimization in childhood, the degree of trait anxiety, and depressive rumination. Furthermore, the prevention and early detection of childhood victimization for its minimization is expected to prevent the future onset of depression, which is socially meaningful towards reducing the number of patients with depression.
